# An All-Atom Model of the Chromatin Fiber Containing Linker Histones Reveals a Versatile Structure Tuned by the Nucleosomal Repeat Length

**DOI:** 10.1371/journal.pone.0000877

**Published:** 2007-09-12

**Authors:** Hua Wong, Jean-Marc Victor, Julien Mozziconacci

**Affiliations:** 1 Laboratoire de Physique Théorique de la Matière Condensée, Université Pierre et Marie Curie, Paris, France; 2 Cell Biology and Biophysics, European Molecular Biology Laboratory, Heidelberg, Germany; University of Waterloo, Canada

## Abstract

In the nucleus of eukaryotic cells, histone proteins organize the linear genome into a functional and hierarchical architecture. In this paper, we use the crystal structures of the nucleosome core particle, B-DNA and the globular domain of H5 linker histone to build the first all-atom model of compact chromatin fibers. In this 3D jigsaw puzzle, DNA bending is achieved by solving an inverse kinematics problem. Our model is based on recent electron microscopy measurements of reconstituted fiber dimensions. Strikingly, we find that the chromatin fiber containing linker histones is a polymorphic structure. We show that different fiber conformations are obtained by tuning the linker histone orientation at the nucleosomes entry/exit according to the nucleosomal repeat length. We propose that the observed *in vivo* quantization of nucleosomal repeat length could reflect nature's ability to use the DNA molecule's helical geometry in order to give chromatin versatile topological and mechanical properties.

## Introduction

The nucleus of all eukaryotic cells contains meters of DNA organized by histone proteins into a hierarchical architecture: the chromatin [1], [2]. The chromatin fundamental unit, the nucleosome core particle (NCP), organizes 147 base pairs (bp) of DNA in a 1.7 left-handed superhelical turns around an octamer of four core histones (H2A, H2B, H3, H4)_2_. Nucleosomes are regularly spaced along DNA forming a beads-on-a-string structure, which can in turn fold into a compact 30 nm diameter fiber. A fifth histone type (H1/H5) helps this folding by interacting with DNA at the entry-exit of each nucleosome [3]. This interaction has been observed at the molecular level *in vitro* [4] and induces the wrapping of 20 additional DNA bp to complete the second full turn around each nucleosome. Semi-compact fibers can then be obtained by the apposition of entry and exit DNA linkers [5]. However, this zigzag folding pattern is not compact enough to explain the high density of chromatin as measured *in vivo* in metaphasic chromosomes [6] (6 nucleosomes/11 nm compared to 11 nucleosomes/11 nm).

Recently, highly compact fibers have been reconstructed *in vitro*, using a strong nucleosome positioning DNA sequence, a high divalent cation concentration, and linker histones [7]. These compact fibers have been imaged by electron microscopy (EM), but the obtained images do not give precise information on the positions of nucleosomes and DNA linkers. In order to provide a model of the internal structure of the compact fiber, Robinson *et al.* measured the dimensions (diameter and linear compaction) of fibers reconstructed with different DNA linker lengths, ranging from 10 to 70 bp. The three main models of fiber internal structure, reviewed in [8] predict discriminant variations of those two dimensions with respect to a change of nucleosomal repeat length (NRL):

- The solenoidal model [9] predicts no variation of the two dimensional parameters with the NRL.
- The two-start super-coiled model, or twisted ribbon [10] predicts a linear variation of the compaction with the NRL.
- The two-start twisted model, or cross-linker model [11] predicts a linear variation of the diameter with the NRL.

**Figure 1:**
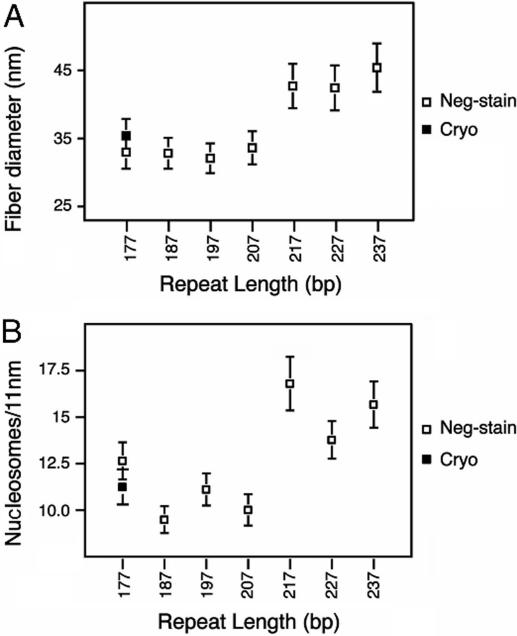
EM-measurements of the diameter (A) and the compaction (B) of reconstituted chromatin fibers containing linker histone for 7 different NRL. Adapted from [7].

The results of Robinson *et al.* are reported on Fig. 1. They find that there are two different classes of fibers. Arrays with NRL ranging from 177 up to 207 DNA bp fold into a fiber that is roughly 35 nm in diameter and 11 nucleosomes/11 nm in compaction, while arrays with linker length ranging from 50 up to 70 bp produce fibers of 45 nm in diameter and 15 nucleosomes/11 nm in compaction. Those measures led them to propose an interdigitated solenoidal structure closely related to the one proposed by Daban and Bermudez [12]. This structure combines two interesting features: it is compact enough to account for the EM measurements and its diameter is fixed by nucleosome face-to-face contacts and therefore does not depend on linker length. Nucleosomes are radially positioned around the fiber axis, the n+5^th^ being stacked on the n^th^. Linkers are bent, making a loop in between two consecutive nucleosomes. The size of this loop is then increasing with the length of the linker. However, in their model, Robinson *et al.* do not provide any explicit path of the DNA linker within the fiber.

In this study, we propose the first all-atom structural model of the chromatin fiber containing H5 linker histone. In the first part of our work, we provide a classification of the different possibilities for nucleosome core particle (NCP) positions and orientations in the context of a homogeneous fiber. We then connect those NCP with DNA linkers of given lengths using skeletal animation together with inverse kinematics (see Material and Methods). For each NRL used in the experiment by Robinson *et al.* we screen all possible nucleosomes positions and orientations compatible with the fiber dimensions. We selected the ones in which DNA linkers were able to connect consecutive nucleosomes without steric clashes in-between atoms or chemical bonds significant distortions. H5 was then placed into the structures that satisfy both energetic and steric constraints according to previous experimental and computational results.

Our findings suggest that, as previously proposed [3], [5], H5 leads to the compaction of the fiber by closing two turns of DNA around the nucleosome. The unexpected outcome of our study is that the chromatin fiber containing linker histones is a highly polymorph structure, tuned by the NRL. Most of the structures we describe here have already been proposed based on numerous experimental and theoretical studies on the 30 nm chromatin fiber: solenoids [9], 2-start helix [8], [11], [13], 3-start helix [14] and 5-start helix [12]. In the past decades, so many different models were proposed for compact fibers that the question whether there is really such a structure ultimately arose [15]. We interpret this multiplicity of models as a property of the fiber itself: there is a multiplicity of fiber structures! We propose that these different structures, which differ both in mechanical [16] and topological [17] properties, are selected by changing the NRL. Those different physical properties in turn can control the accessibility to the DNA sequence.

## Results

### Inventory of NCP positions within a regular fiber

The input data used in our modeling process are the dimensions (i.e. diameter and compaction) of the chromatin fiber. Very regular and homogeneous fibers have been reconstructed and carefully measured, *in vitro*, using a strong positioning sequence, linker histones and a high magnesium concentration [7]. Using cryo-EM results, a technique that is more likely to keep the structures intact, it has been found that the diameter of the fiber was ∼35 nm. This diameter was conserved for NRL comprised between 177 and 207 bp. For longer NRL, the diameter was shown to jump to ∼45 nm. In a similar manner, the compaction of such a fiber was shown to increase from 11 nucleosomes/11 nm for 35 nm diameter fibers to 16 nucleosomes/11 nm for 45 nm diameter fibers (Fig. 1). We therefore based our *in silico* fiber reconstruction on those two values of the diameter and compaction.

**Figure 2:**
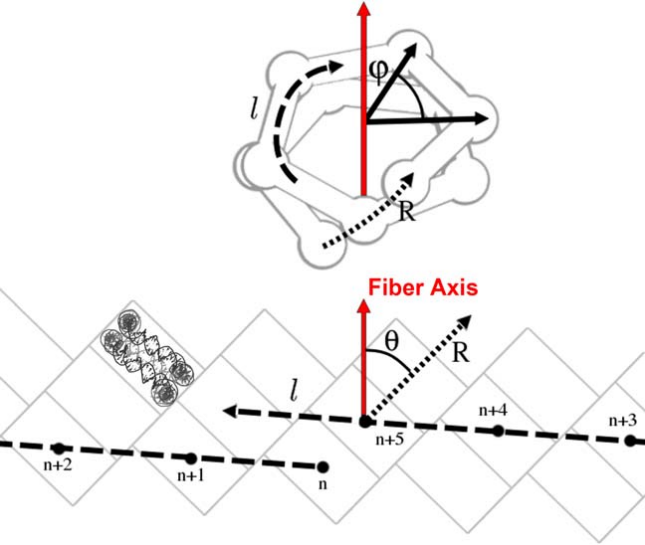
Scheme of the NCP positions within the chromatin fiber. Top: the spheres represent 3D positions of the nucleosomes' centers of mass. The joints in between them show the DNA connectivity. The j angle is the azimuthal rotation angle in between consecutive nucleosomes. Bottom: schematic representation of the cylindrical surface containing all nucleosomes' centers of mass. The angle q is the nucleosomes tilt. A scaled all-atom structure of nucleosomal DNA has been included in one of the box representing nucleosomes. The lower case l corresponds to the left-handed helicity of the DNA path connecting nucleosomes. The capital R corresponds to the right-handedness of the helices made by stacked nucleosomes. This fiber is therefore denominated 5Rl in our classification.

The first step of our modeling process is to place 30 NCP all-atom structures in 3D space in order to obtain a regular fiber of given diameter and compaction (see Material and Methods). Previous structural studies on isolated NCP showed that they have a strong tendency to interact through their faces [18], forming arcs, helices [19] or liquid crystal phases [20]. There is now increasing evidence that this stacking is also favored within dense chromatin fibers [8], [13]. Additionally, EM [21] and theoretical [16] studies pointed out that in the context of a chromatin fiber, nucleosomes are radially distributed, with their dyad axis orthogonal to the fiber axis [21]. Based on these experimental pieces of evidence, a simple space-filling model suggests that regular fiber structures are multiple left or right-handed, helices of stacked nucleosomes [12]. To label these different structures, we used one number and two letters. The number refers to the number of starts of the helix while the first letter, capital R or L, refers to the handedness of the helix and the second letter, lower case r or l, refers to the handedness of the DNA path between nucleosomes (Fig. 2). We would like to stress here that all fiber models with a DNA linker placed inside the fiber and stacked nucleosomes fall into this classification. For example, the two-start helix proposed by Richmond and colleagues from the crystal structure of a tetra-nucleosome [13] is the 2Lr fiber whereas the one proposed by Robinson and co-workers is the 5Rl [7]. Importantly, the authors of the latter paper refer to their structure as a one-start structure (according to the DNA path around the fiber axis), whereas in our notations, it is a five-start structure, the n+5^th^ nucleosome being stacked on the n^th ^one.

Based on this classification, we built a library of 16 template fiber structures ranging from one to five start helices, by placing the 30 NCP crystal structures (1KX5) [22] according to the measured fibers dimensions. We then tried to connect those NCP with DNA linkers of different lengths, corresponding to the different NRL used in the experiment and selected the fibers in which consecutive NCP can be connected without steric clashes in-between atoms or chemical bonds significant distortions.

### Placement of the DNA linkers connecting consecutive NCP

We found that the phasing angle between DNA at the exit of one nucleosome and the entry of the consecutive nucleosome is a very strong constraint to select a structure, due to the DNA natural helical twist [16], [23], [24]. In fact, for each NRL, only 1 to 3 NCP positions and orientations were compatible with both the length and the relaxed twist of the linkers (see Table 1). In some cases, phasing and distance between consecutive NCP were compatible with a given NRL, but linkers were intersecting with each other. Using forward kinematics on the first 20 bp at the entry/exit of the nucleosomes, i.e. by moving them by hand, we found that wrapping 20 bp of the linker around the histone octamer was a very efficient way to avoid this steric hindrance.

**Table 1:** Possible fiber structures for different NRL.

Start	Fiber Helicity	DNA Path Helicity	Diameter 35,4 nm					45,4 nm		
			177	187	197	207	217	217	227	237
1	R	N/A*								
										
	L	N/A								
										
2	R	N/A				X				
										
	L	N/A								X
										
3	R	r			X					
		l			X					
	L	r								
		l							X	
4	R	r								
		l		X				X		
	L	r								
		l								
5	R	r		X				X		
		l	X	X						
	L	r								
		l								
									

Left entries: all different fiber structures as described in Fig. 2. Top entries, different repeat length and different diameters of the fiber, taken from [7]. When we found that a structure is compatible with a NRL, we placed an X in the corresponding cell.

* Not Applicable

**Figure 3:**
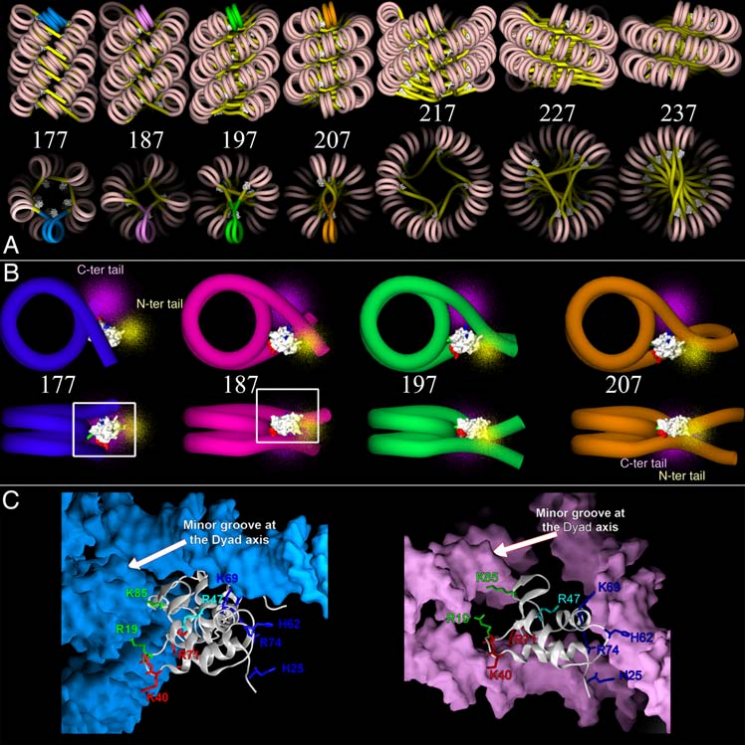
Structures of the chromatin fiber for different NRL. A) All-atom models of the 7 chromatin fibers obtained by H5-induced folding of a regular chromatin array [7]. For clarity, DNA is shown as a tube. The yellow color corresponds to linkers whereas nucleosomal DNA is presented in pink. Molecular surfaces of H5 globular domains are shown in white. Each structure corresponds to a different NRL. From left to right: 177 bp, 5Rl; 187 bp, 4Rl; 197 bp, 3Rr; 207 bp, 2Rl; 217 bp, 4Rl; 227 bp, 3Ll; 227 bp, 2Lr. For 177 to 207 bp NRL, one DNA repeat length is shown in color (blue, purple, green and orange for 177, 187, 197, and 207 bp NRL respectively). B) Close-up of the four chromatosomes presented in color in A. C- and N-terminal tail extensions are presented in light pink and yellow respectively. The H5 globular domain residues that interact with entry and exit DNA linkers are colored in blue and red. The residues that are interacting with nucleosomal DNA at the dyad axis are colored in green. C) Molecular details of the GH5 positioned on a nucleosome (for 177 and 187 bp NRL). The residues involved in DNA binding [25]–[27] are shown in licorice. In blue: residues interacting with one DNA linker; in red: residues interacting with the other DNA linker; in green: residues inserted into the DNA major groove next to the dyad axis. R47, shown in cyan is never found in contact with DNA.

For the 177 bp NRL, the only structure that satisfied all the geometric constraints had nucleosomes stacked in a 5-start right-handed helix and DNA following a left-handed path around the fiber axis: according to our classification, it is a 5Rl. This structure is very close to the structure proposed by Robinson *et al.* based on simple geometric constraints. In their paper, they suggested that longer linkers (for NRL 187, 197, 207 bp) were bent to fit in the interior of the fiber. Our modeling procedure allowed us to test this prediction. We found that this structure was still compatible with a 187 NRL. However, for longer NRL, 5Rl type fibers could not be obtained anymore because of persistent steric clashes in between linkers. For 197 and 207 bp NRL, only 3 and 2-start structures could match physical and geometrical constraints. For the 217 bp NRL, we found no structure compatible with a 35 nm diameter fiber (Table 1; 35 nm diameter, 217 bp NRL). However, if the diameter was increased by 10 nm, as suggested by the EM measurements [7], a new class of fibers could be obtained. The 217 bp NRL was compatible with both 4 and 5-start right handed helices whereas 227 and 237 bp NRL were compatible with 3 and 2-start left handed helices. For each NRL, a 3D representation of the DNA path in the compact fiber of lower energy is presented in Fig. 3A. As can be seen from the top views, DNA follows a path shaped as: a pentagon for 177, a square for 187 and 217, a triangle for 197 and 227 and a line along the diameter for 207 and 237. This simple geometrical fact can explain how linkers of different lengths can fit inside an internal cavity of constant size.

### Position, orientation and role of linker histone within the chromatin fiber

The last step of our modeling was to place the linker histone globular domain inside our “DNA-and-core-histones” all-atom fiber. Several models have been proposed for the placement and orientation of H1/H5 [3]. The common feature of those models is that H1/H5 binds at the entry/exit of the nucleosome and closes two turns of DNA around the core histones by interacting with one or two DNA linkers. Biochemical studies have identified the positively charged surface residues involved in DNA-H1/H5 contacts [25]–[27]. Recently, using a computational approach, plausible positions and orientations of the H5 linker histone on a nucleosome were proposed [28]. The residues involved in this positioning, i.e. those interacting with DNA, were matching the residues pointed out by former biochemical studies. Here, we placed the atomic structure of the globular domain of H5 (1HST) [29] according to these published structural data.

In Fig. 3B we present a detailed view of chromatosomes (i.e. nucleosome+linker histone) found in fibers constructed for NRL equal to 177, 187, 197 and 207 bp. In these four structures, the globular domain of H5 could bind three DNA double strands. Our model showed different conformations of the chromatosome for different fiber structures, suggesting that H1/H5 helps adjusting the entry/exit angle of DNA on a nucleosome for all the different possible fiber structures.

First, two different positions of the globular part of the linker histone GH5 within the chromatosome could be identified:

- For 177 bp repeat length, the entry and exit linkers were fully wrapped on the nucleosome and GH5 could be placed on the dyad axis, just in between the two linkers, in a similar way as in the former model of Allan *et al* [30] (Fig. 3B, dark blue).
- For longer NRL, the entry/exit linker are more opened and form a pocket in which GH5 can be placed, bridging both DNA linkers together with the nucleosomal DNA at the dyad axis (Fig. 3B, purple, green and orange).

In Fig. 3C, we present a detailed view of the contacts made by GH5 with DNA in the chromatosome for those two conformations. Importantly, 80% of the contacts in between GH5 and DNA were the same in both conformations. By comparing those two conformations, we propose that a first tuning of the entry/exit linker angle can be achieved via a ball-bearing-like rotation of GH5 on itself around the dyad axis.

An examination of the DNA linker path at the exit of each chromatosome pointed out that a second way to tune the entry/exit angle was to make a stem in between linkers at the chromatosome exit (see Fig. 3B). Similar structures were indeed observed on mono-nucleosomes assembled on linear DNA fragments together with linker histones [31] and on regular fibers [5]. Different stem lengths could account for the differences in DNA linker orientations in 4, 3, and 2-starts structures. The H5-dependent organization and dynamics of the nucleosome entry/exit DNA on mini-circles [32] pointed out the role of the linker histone C-terminal tail in stabilizing such structures. Accordingly, in our model, both the C-terminal and N-terminal tails of the linker histone were found to be in close contact with the DNA linkers at the entry/exit of the chromatosome. We propose that this interaction depends on linker length:

- For the 177 bp NRL, the DNA joining consecutive chromatosomes is only 10 bp long. There is no possible binding site for H5 tails and the linker is essentially straight.
- For the 187, 197 and 207 bp NRL, H5 tails are able to make respectively, one, two, and three contacts with DNA linkers. These interactions induce DNA kinks, and a consequent change in DNA linkers' relative direction at the chromatosome exit.

## Discussion

### Linker-linker and nucleosome-nucleosome interactions in a compact chromatin fiber: energetic considerations

In the fibers presented on Fig. 3A, 60 to more than 80% of the fiber volume is occupied by proteins or DNA. Linkers are aligned next to each other allowing this very high fiber compaction. This arrangement is reminiscent of DNA cholesteric liquid crystals (especially the structures obtained for the 197, 207, 227 and 237 bp NRL). The pitch of the cholesteric phase depends on the number of starts of the chromatin fiber helix and ranges from 35 to 100 nm. These values are in good agreement with *in vivo* measurements in sperm nuclei [33]; however, whereas DNA cholesteric crystals are only left-handed, our model shows both left and right-handed helicity. The distance between two DNA double strands can be found to be as small as 2.5 nm, as observed in hexagonal packing of bacteriophage DNA [34]. It should be noticed that this high compaction is favored by the strong positive divalent ion concentration. Mg^2+^ ions not only permit negatively charged DNA strands to come into vicinity, but moreover participate in the formation of electrostatic bridges which in turn insure the stability of the structure [35]. When the NRL increases from 177 to 207 bp, the amount of DNA material inside the fiber also increases. We propose that the concentration of divalent cation is locally rising and participates in the electrostatic stability of the structure.

**Figure 4:**
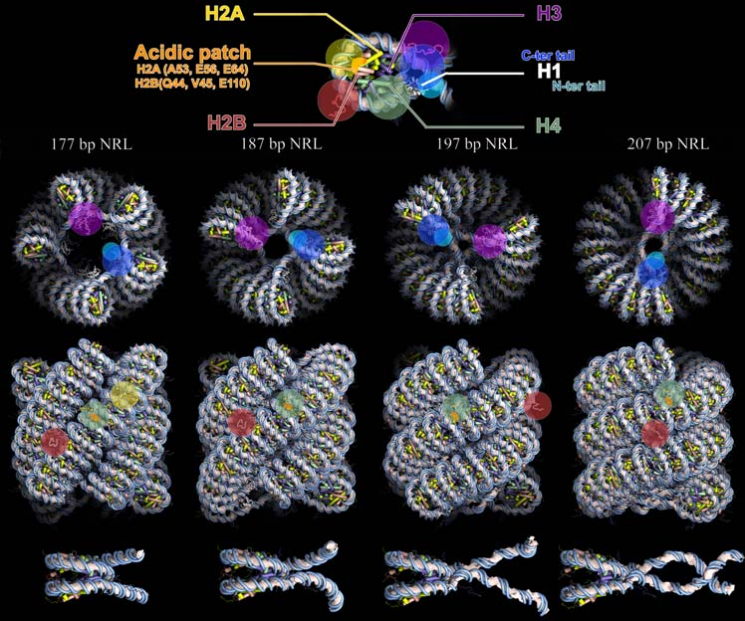
All-Atom structures of the chromatin fiber for 4 different NRL. Top: one nucleosome is presented at higher resolution to show the color codes used for histone, histone tails and specific residues. Histone tail extensions are presented as colored circles. Middle: top and side view of the all atom fibers for the 177, 187, 197 and 207 bp NRL. Bottom: close-up on each chromatosome highlighting the gaping of each nucleosome.

The stacking of nucleosomes bring into close proximity residues H2A–(A53, E56, E64), H2B–(Q44, V45, E110) of one nucleosome with H3–(E73, Q76, D77), H4–(K20, L22, R23) of the neighboring nucleosome so that specific interactions between them can occur upon nucleosome gaping [36] and potentially lock the fiber in a compact state [8], [22] (see Fig. 4). Our model does not include explicit histone tail rearrangements. However, since we provide a detailed model of the dense fiber, the role of each histone tails in nucleosome/nucleosome and linker/nucleosome interactions can be addressed. On Fig. 4, we present the extension of each N-terminal tail and H2A C-terminal tail based on results obtained using a mesoscopic oligonucleosome model [37]. H3 tails interact with DNA linkers; H4 and H2A tails participate in nucleosome stacking interactions whereas H2B tails are likely to bridge adjacent columns of stacked nucleosomes. We hope that this model will help to gain meaningful insights into the influence of histone tails modifications on chromatin structure.

### *In vivo* relevance of our results

Our approach intends to model fibers obtained *in vitro* in very specific and controlled conditions. The question of the *in vivo* relevance of our modeling therefore arises.

In our models, the nucleosome tilt angle is ranging from 12° for 2-start helices to 40° for 5-start helices. This value is in good agreement with the range measured for chromatin fibers isolated from different species (13° to 38°) [7]. We suggest that the wide range of tilt angle values observed *in vivo* corresponds to different structures rather than to an error around a mean value corresponding to a unique structure.

While comparing the radius of isolated chromatin fibers with the NRL in different organisms, Langmore and co-workers, concluded that there is a linear relationship between those two quantities [11], in apparent contradiction with the *in vitro* results found by Robinson *et al.* [7]. However, a careful re-examination of their results leads to an opposite conclusion. In fact, in Fig.7 from reference [11], one can clearly distinguish two classes of fibers, one of them, for NRL shorter than 210 bp, corresponds to fiber diameters around 31 nm and the other, for longer linkers, corresponds to fiber diameters around 39 nm. The fact that two statistically different clusters exist suggests that the two different classes of diameter reported in [7] can be relevant *in vivo* as well. However, note that difference in values (31 nm vs. 35 nm and 39 nm vs. 45 nm) for the compaction and diameter of the fiber found in different studies can be explained by the different biological sample preparation and imaging.

**Figure 5:**
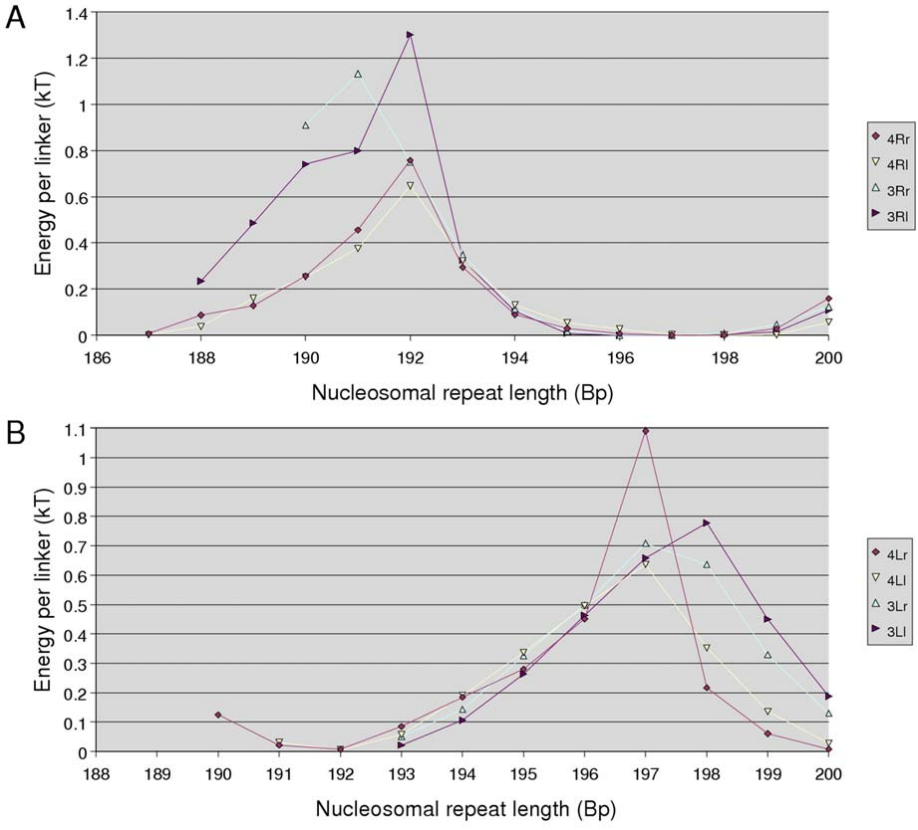
Plots of DNA linker twist for different structures and NRL. A) For right-handed helices. B) For left-handed helices. Non-displayed points correspond to NRL for which the linker does not join the consecutive nucleosomes.

It is still a matter of debate whether nucleosomes are strongly and regularly positioned *in vivo*. To see how our model can account for non-uniform NRL, we plotted the twist energy of the linkers for all NRL ranging from 186 to 200 for all possible structures (Fig.5). As expected, for right-handed helices, minimal energy is found for 187 and 197 bp NRL. This minimum is rather flat allowing 1 to 2 bp local changes in the NRL without energy increase.

Fig. 5 also shows that for left-handed helices the minimum energy is found for 192 bp NRL. Interestingly, it has been measured that 192 bp NRL favors the formation of left-handed helices [11] and that chromatin fiber isolated from chicken erythrocyte is a mix of both left and right handed conformations [21]. This further suggests that the different structures reported here might be relevant *in vivo.*

The NRL used by Robinson *et al*. in their experiment (177, 187, 197, 207, 217, 227 and 237 bp) [7] were chosen to match the NRL most often found in nature (159, 171, 178, 188, 192, 196, 206–207 and 217–218 bp) [23]. As the period of this NRL quantization is close to the helical repeat of DNA, it has been proposed to be required for selecting a precise phasing angle between consecutive nucleosomes, necessary for higher order folding of the beads-on-a-string nucleosomal array. This point was illustrated in [32] in the case of the solenoidal model and could be extended to other structures. Our results support a similar, however, more precise explanation: we propose here that different NRL correspond to different structures, ranging from the classical solenoid for 167 bp (not shown) to n-start cross-linker models. This prediction could be tested by inter-nucleosome cross-linking followed by linker digestion experiments on regular fibers as illustrated in [8].

## Materials and Methods

### Calculation of NCP positions within a fiber

The first step of our modeling is to place the NCP, in a way that prevents steric clashes (Fig. 2). Knowing the dimensions of the fiber and the number of starts considered, the tilt *θ* of the NCP with respect to the fiber axis can be simply calculated:
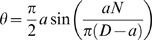
(1)In this expression, *D* is the diameter of the fiber, *a* is the diameter of a NCP, and *N* is the number of starts of the helix.

The position of the n+1^th^ NCP can be derived from the position of the n^th^ one by rotation around the fiber axis of an angle *ϕ* and a translation of length *d* along the fiber axis
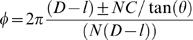
(2)

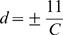
(3)where *C* is the compaction expressed in nucleosomes/11nm. The sign±in the expression of *ϕ* corresponds to right or left handed helices (R or L), while it corresponds to right or left-handed DNA paths (r or l) in the expression of *d*.

Knowing the diameter and the compaction of a fiber, we can place the atomic structure of each nucleosome (1KX5) [22] according to those geometric constraints (Fig. 2).

### Molecular modeling of linker DNA

**Figure 6:**
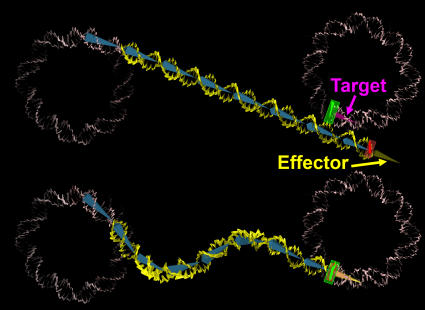
Illustration of the IK chain used in order to place linkers in between consecutive nucleosomes. In pink we show the nucleosomal DNA and in yellow the DNA linker. The bones supporting the linker DNA are shown in blue. We start from a straight conformation and then try to see whether or not it is possible to superimpose the last bp of the DNA linker (in red) with the first DNA bp of the consecutive nucleosome (in green) when relocating the effector on the target.

We first remove 10 DNA bp at the entry-exit of each NCP. We then add a straight, B-form, random sequence, DNA double helix of (NRL–127) bp length at the exit of the first nucleosome. This DNA is created with a rise of 3.4 Å and a helical repeat of 10.5 bp using JUMNA [38]. At the end of this linker, we add one extra base pair (in red on Fig. 6), which we try to align with the entry of the consecutive nucleosome (in green on Fig. 6) effectively superimposing their coordinate frames. To achieve this goal, we place a linear skeleton along the DNA axis divided in bones of 10 bp length (in blue on Fig. 6) and use inverse kinematics (IK) in order to deform this skeleton. IK is common in robotics and has already been used in molecular modeling studies of protein loops [39]. Solving the IK problem tells you how much you have to rotate each joint in an articulated chain so that the end of the chain (effector on Fig. 6) reaches a required position and orientation (target on Fig. 6). Once the skeleton has been deformed, it is possible to reconstruct the full DNA double helix around it using deformation matrices. The all-atom structure is then refined using a short energy minimization run. The novelty of this approach is that it offers the possibility to build all-atom models of bent DNA linkers. We made use of the free 3D package Blender (http://blender3d.org/cms/Home.2.0.html) because it offers a robust IK algorithm based on a damped Jacobian transpose method [40] completed with a constraint system. The constraint system allows us to limit the rotational freedom of each link in order to mimic the shape a real DNA would adopt. For each linker, we restrict the torsion/bending of DNA to 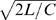 where L is the length of the linker and C the respective twist/bending persistence length of DNA (50 nm/75 nm). This allows us to select structures in which the DNA linker joins consecutive nucleosomes with bending and twisting energies smaller than kT. Energies have been calculated as follows:
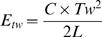
where ***C*** is the twist persistent length of DNA, ***Tw*** the twist of the linker and ***L*** the linker length. Note that we increased the bending flexibility of the first 20 DNA bp at the entry/exit of each nucleosome, to mimic its interaction with H5 or with histone core tails. When we find a linker fitting inside the structure, we simply replicate it in order to form a continuous DNA double strand connecting all nucleosomes in the fiber.

### Docking of linker histone on the nucleosome

We first minimize the atomic structure (1HST) and run a 800 ps molecular dynamics simulation in explicit solvent using GROMACS [41] in order to relax the protein side chains. Then we insert lysine K85 next to the major groove of DNA at the dyad axis, as it was found to be in 27 out of 30 top-ranked docking positions [28], and oriented the globular domain so that the residues involved in DNA binding (R42, R47, R73, R74, R94, K40, K69, K85, K97, H 25 and H62) are making close contacts with the entry and exit linkers.
